# *Salmonella typhimurium* Endocarditis and Myocarditis in a Cat

**DOI:** 10.1155/2019/7390530

**Published:** 2019-12-09

**Authors:** Andrea Vercelli, Enrico Lo Cicero, Luca Pazzini

**Affiliations:** ^1^Clinica Veterinaria città di Torino, Corso Traiano 99/d, Torino 10135, Italy; ^2^La Vallonea laboratorio di analisi Veterinarie, Via Giuseppe Sirtori 9, Rho, Milano 20017, Italy

## Abstract

An 8-month-old neutered male outdoor cat was brought to our surgical center for a sudden onset of diarrhea, pyrexia, and lethargy. Physical examination revealed a loud left parasternal systolic murmur with no thrill. An echocardiogram showed a large hyperechoic vegetation (about 9 mm thick) on the aortic valve leaflets. The results of Doppler ultrasound examination were compatible with severe aortic stenosis. A singular urine culture test performed by cystocentesis samples enabled the isolation of more than 10^5^ CFU/ml in a pure culture of *Salmonella typhimurium*. Enlarged mesenteric lymph nodes and moderate dilatation of small bowel loops were found on abdominal ultrasound examination. The patient was treated with marbofloxacin (2 mg/kg IM every 24 hours), cefazoline (20 mg/kg SC every 12 hours), metronidazole (10 mg/Kg IV every 12 hours), clopidogrel (18.75 mg PO every 24 hours), atenolol (0.5 mg/kg OS every 12 hours), and fluid therapy (ringer acetate 2.5 ml/kg/h), but after three days in hospital the patient died from presumed septic shock. A urine culture revealed that *Salmonella typhim*urium was sensitive to third generation cephalosporins but not to fluoroquinolones. Necropsy, histologic examinations, culture of the aortic valve, and PCR analysis of the aortic valve leaflets were eventually performed and *Salmonella typhimurium* endocarditis with myocardial phlegmon was confirmed. Endocarditis is a rare disease in cats and poorly described in the veterinary literature. To the best of the authors' knowledge, this is the first report of *Salmonella typhimurium* endocarditis and myocarditis in a cat.

## 1. Introduction

Feline infective endocarditis is a rare inflammatory disease of the valvular endocardium and occurs when microorganisms that come from other parts of the body, such as the oral cavity, gastrointestinal tract, urogenital tract, and skin, penetrate the bloodstream and reach the valvular or rarely the parietal endocardium of the heart [1, 2].

The predisposing factors of feline infective endocarditis are endothelial damage due to congenital valvular diseases, intravenous catheterization, immunosuppression, and infection with virulent microorganisms. The main causative agents are *Staphylococcus aureus*, *Escherichia coli*, *Streptococcus* sp., and *Bartonella henselae *[1, 3]. *Salmonella typhimurium* has never been described before in feline patients. The bacilli of the genus Salmonella are present in the environment, in the soil, and in the water, and can be found as commensal or pathogens in the intestine of animals such as mammals, birds, reptiles, and humans [4]. *Salmonella typhimurium* is a typical food-borne pathogen. In cats, the feeding of raw poultry or contaminated food or infected wild birds or mice, can lead to Salmonellosis. Susceptibility to disease is highest in immunosuppressed cats, young animals, pregnant, and those with underlying immunosuppressive illness such as retroviral infection (FIV or FeLV), neoplasia, diabetes mellitus or immune suppressive drug therapy [4–6]. The clinical symptoms and signs of feline infective endocarditis are the consequence of four simultaneously active mechanisms: (1) the valve damage (valvular stenosis or valvular regurgitation) with subsequent hemodynamic consequences of pressure or volume overload; (2) the severity of infection; (3) thromboembolism; and (4) immunological alterations [1–3]. The consequences of the infection are related to the toxicity of the microorganisms and to the intensity of their propagation to the various organs; the embolic manifestations, depending on the friability of the valve vegetations, particularly affect some anatomical regions (e.g., the brain, limbs, kidneys, and spleen); the immunological phenomena are the consequence of the stimulation of the immune system by pathological agents, with formation of immune complexes in the joints, in the uvea, and in the renal glomeruli inducing respectively to arthritis, uveitis, and glomerulonephritis [1–3]. Feline infective endocarditis should be suspected in patients with fever and no obvious infectious focus, particularly if a heart murmur is present. Respiratory distress and locomotor abnormalities (lameness or paralysis) are common presentations in feline patients [1, 3]. The echocardiogram, the blood cultures, and the PCR for Bartonella are considered the main diagnostic investigations to reach the diagnosis of feline infective endocarditis [1–3]. The cornerstone of the therapy consists of a targeted antibiotic therapy, in the management of heart disease and in the prevention of embolization, but unfortunately the prognosis is grave and often reserved [1–3].

## 2. Case Description

An 8-month-old outdoor neutered male European domestic short-hair (DSH) cat was referred to our clinic with a 3-week history of diarrhea, anorexia, and lethargy. Physical examination revealed pyrexia (40.1°C). The mucous membranes were moderately pale and the cat was estimated to be 6-7% dehydrated. The body weight was 3.3 kg on initial presentation with a body condition score of 6/9. The peripheral lymph nodes were normal. Abdominal palpation revealed small bowel distention with fluid. The heart rate was 250 bpm with a moderate grade III/VI left parasternal systolic murmur with no thrill; the femoral pulses were weak and synchronous with the heart beats. The respiratory rate was 30 breaths/min, without abnormal lung sounds. No respiratory distress was detected. An electrocardiogram showed sinus tachycardia. Hematological abnormalities included: moderate to severe non regenerative anemia (packed cell volume 20.8%, reference range 30.3–52.3%), absolute aggregate reticulocyte count was 38 × 10^9^/L (regenerative response > 80 × 10^9^/L), neutrophilia 13.2 × 10^9^/L (reference range 4.8–10.29 × 10^9^/L) with a left shift, monocytosis 0.70 × 10^9^/L (reference range 0.05–0.67 × 10^9^/L) and pseudothrombocytopenia 88 × 10^9^/L (reference range 151–600 × 10^9^/L) due to platelet clumping. Biochemical abnormalities included elevation of: creatine kinase 515 U/L (reference range 91–326 U/L), blood urea nitrogen 67 mg/dl (reference range 18–41 mg/dl), aspartate aminotransferase 46 U/L (reference range 21–44 U/L), total protein 8.71 g/dl (reference range 5.5–7.7 g/dl).

**Figure 1:**
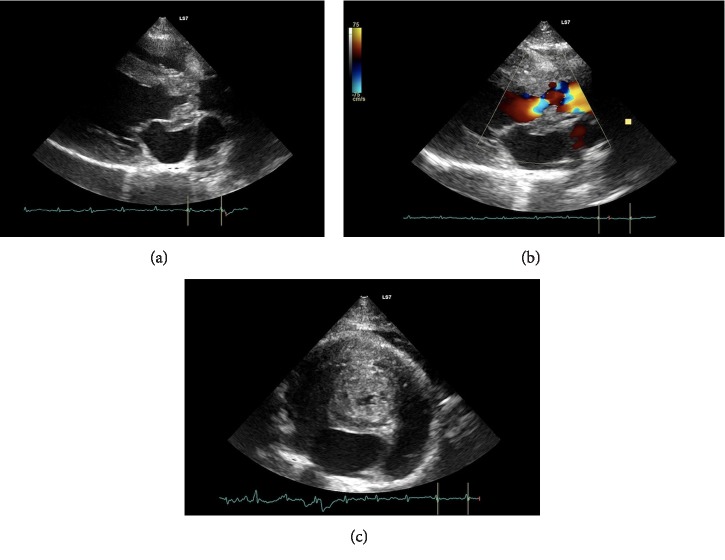
The right parasternal long axis view optimized for the left ventricular outflow tract, shows severe thickening of the aortic valves compatible with aortic bacterial endocarditis (a). In the right parasternal long axis view the aortic color doppler shows a significantly turbulent flow at the valvular cusps, suggestive of valvular aortic stenosis due to bacterial endocarditis (b). The right parasternal transverse (short axis) heart base view depicts, in the middle of the sector image, dramatic thickening of the aortic valve cusps (9.8 mm). The left atrium is below the aorta and it appears slightly compressed by the inflammatory process. On the upper right portion of the picture there is slight anechoic pleural fluid (c).

Concurrent hypoalbuminemia was found to be 2.78 g/dl (reference range 3–4.6 g/dl). The electrolyte levels were low: total calcium 2.07 mmol/L (reference range 2.1–2.87 mmol/L), sodium 142.8 mmol/L (reference range 150–165 mmol/L), and potassium 3.13 mmol/L (reference range 3.5–5 mmol/L). The iron level was 7.16 mol/L (reference range 10.7–37.6 mol/L). Thoracic radiography (3 views) showed no abnormalities in the cardiac silhouette, lung fields, and the pulmonary vessels. An echocardiogram (2-dimensional, M-mode, and Doppler) was performed in our clinic by V. A. using a 4–8 MHz phased-array transducer; the patient was not sedated. The 2-dimensional study revealed a large hyperechoic vegetation of the aortic valve leaflets (9 mm, on the right parasternal long axes, 5 chamber view) (Figure 1).

The mitral, tricuspid and pulmonic valves were normal, M-mode the left ventricular internal dimension at end-diastole was 18.4 mm [15.1 mm ± 2.1] [7] and the left ventricle internal diameter at the end-systole was 12.9 mm [6.9 mm ± 2.2] [7]. The interventricular septum at end-diastole and end-systole showed remarkable thinning: 3.3 mm [5 mm ± 0.7] [4] and 6.1 mm [7.6 ± 1.2] [7], respectively (M-mode study). The left ventricle caudal wall thickness was normal at end-diastole and end-systole: 4.6 mm [4.6 mm ± 0.5] [3] and 7.1 mm [7.8 mm ± 1] [7], respectively (2-dimensional study). Global left ventricular contractility was mildly depressed and fractional shortening was 29.6% [40% ± 10%] [7] (M-mode study). The left atrial diameter at 9 mm was below the reference range [12.1 mm ± 1.8] [7] and the ratio of the left atrial dimension to the aortic annulus dimension was 0.9 [1.29 ± 0.23] [7] (2-dimensional study). There was a significant Doppler increase of the aortic flow velocity on subcostal view: aortic velocity 4.79 m/s [1.1 ± 0.2] [7] equivalent to an aortic pressure gradient of 91.9 mmHg. After performing an echocardiogram, the differential diagnosis of valve vegetation included infectious endocarditis, sterile granuloma, fungal granuloma, tumor, and thrombus. Other clinical signs, such as fever and gastrointestinal signs, suggested that the patient was much more likely to have endocarditis than a thrombus. Abdominal ultrasonography revealed enlarged mesenteric lymph nodes and moderate dilatation of small bowel loops. The patient was hospitalized and antibiotic treatment was started with metronidazole (10 mg/kg IV every 12 hours), cefazoline (20 mg/kg SC every 12 hours), and marbofloxacin (2 mg/Kg IM every 24 hours).

Atenolol (0.5 mg/kg PO every 12 hours) was administered in view of the severity of aortic valve stenosis and to prevent fatal ventricular arrhythmias, but considering the concurrent mild systolic dysfunction, arterial blood pressure and fractional shortening were assessed twice daily. Clopidogrel (18.75 mg PO every 12 hours) was administered for the prevention of cardiogenic embolism. During hospitalization the patient was rehydrated by intravenous infusion of isotonic crystalloid solution (Ringer's acetate, 2.5 ml/kg/h). After 48 hours lactate was increased to 3.8 mmol/L (<1.46 mmol/L) and after 72 hours the patient developed hypothermia, bradycardia, low systolic blood pressure 85 mmHg (>120 mmHg), and, finally, cardiac arrest. Blood polymerase chain reaction for *Bartonella henselae *and a urine culture test were performed.

The urine culture test showed growth on MacConckey Agar and Brilliance^TM^ Salmonella Agar base (Oxoid®, UK) between 10^5^ and 10^6^ UFC/ml. The organism was biochemically identified as *Salmonella typhimurium* with the use of API 20E® and API 50CHL® (bioMerieux®, France). The serotype was assessed using polyvalent Salmonella Antisera (Oxoid, UK) for somatic (O) and flagella antigens (H). Antibiotic susceptibility testing was assessed with Kirby–Bauer method (CLSI), and it showed that *Salmonella typhimurium* was sensitive to third generation but not to fluoroquinolones, blood polymerase chain reaction for *Bartonella henselae *was negative, serology for FIV/FeLV was not performed for lack of owner's consent.

**Figure 2:**
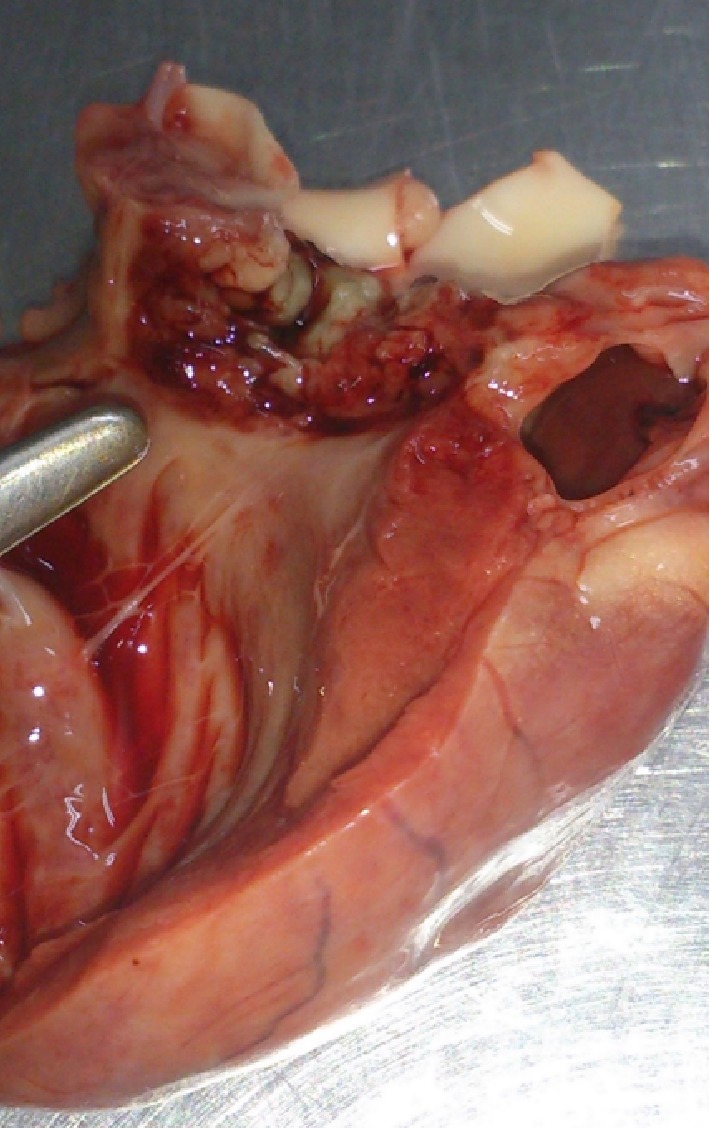
Heart necroscopic findings. Aortic valvular suppurative endocarditis with focal myocarditis. Note the large vegetation on valve leaflets.

A necropsy confirmed the presence of a large vegetation (9 mm in diameter) on the aortic valve leaflets (Figure 2).

**Figure 3:**
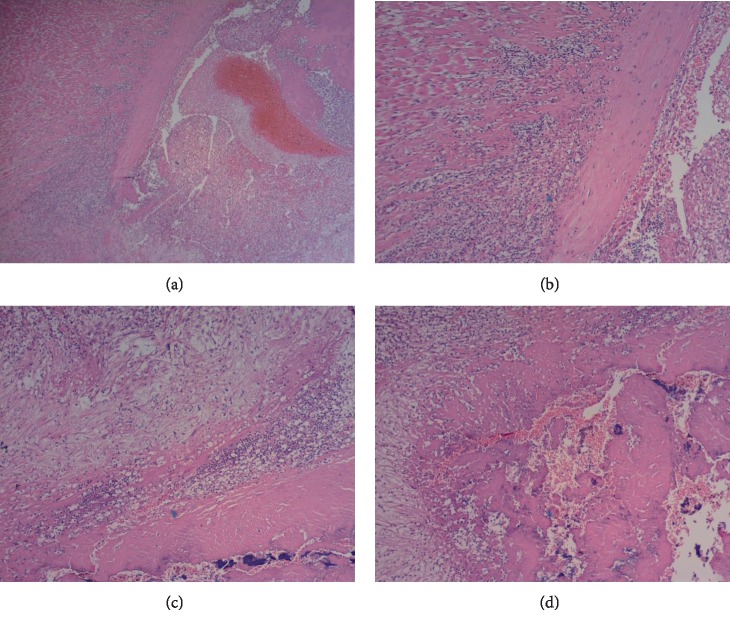
Aortic valve leaflets and myocardium are depicted. Severe diffuse acute suppurative endocarditis and myocarditis is shown (a, original magnification 10x). Separating, surrounding and replacing cardiomyocytes, there are numerous degenerate karyorrhectic neutrophils, a moderate number of erythrocytes, eosinophilic cellular, and karyorrhectic debris (necrosis) and small, basophilic, rod-shaped, 0.5 × 1 *µ*m bacterial aggregates (b, c, d, original magnification 40x) (hematoxylin and eosin stained, formalin fixed-paraffin embedded tissue sections).

Histology of the aortic valve leaflets and myocardium was consistent with neutrophilic endocarditis and suppurative localized myocarditis caused by *Salmonella typhimurium* (Figure 3).

Postmortem culture of the aortic valve was positive for *Salmonella typhimurium *at more than 10^5^ CFU/ml; bacterial taxonomy was evaluated by conventional biochemical test (bioMerieux, France) and using polyvalent Salmonella antisera (Oxoid, UK) for somatic (O) and flagella (H) antigens.

## 3. Discussion

Feline infectious endocarditis is a rare disease, with a prevalence of 0.006% to 0.018% in cats [8]. Common bacterial isolates from feline and canine endocarditis include *Staphylococcus aureus*, *Escherichia coli*, *Streptococcus *spp. and *Pseudomonas *spp. [5, 9–11]; *Bartonella henselae *is considered a common infectious agent in feline endocarditis; domestic cats and wild felids are considered its natural reservoir [12, 13]. Infectious endocarditis is rare also in human medicine with an annual incidence rate of about 3–10 per 100,000 population, with *Streptococcaceae* being the most frequently identified microorganism [6, 12, 13]. Enterococcal infectious endocarditis has increased significantly in the past ten years [13–14]. Several cases of endocarditis caused by *Salmonella typhimurium *were described in human medicine [6, 14]. In veterinary medicine, a case of granulomatous myocarditis caused by *Salmonella enterica* subsup. *arizonae* was described in a Dumerili's boa (*Acrantophis dumerili*) [15], but no cases of endocarditis or myocarditis caused by *Salmonella typhimurium *have previously been described in cats. All Salmonella infections are considered zoonoses, except for *Salmonella typhi, Salmonella paratyphi *A, and *Salmonella paratyphi *B, which are human-specific diseases [5]. At the time of the diagnosis the owner had not developed any clinical signs related to the disease; we do not have further data about his health condition. In our case, infectious endocarditis was readily suspected on physical examination at presentation. The clinical examination showed a moderate grade III/VI left parasternal systolic murmur due to severe aortic outflow tract obstruction. The presence of a diastolic murmur at the left heart base, the onset of a new murmur, or a murmur that changes in intensity or characteristics have been considered classic clinical features of valvular endocarditis [9]. Signs of left-sided congestive heart failure are common when the aortic valve is affected [3] but our patient did not show pulmonary congestion or pleural effusion.

Feline patients with infectious endocarditis typically present with clinical signs consistent with cardiac failure and locomotor abnormalities suggestive of either thromboembolic disease or inflammatory arthritis [3]. In our patient, lameness or locomotor abnormalities were not clinically obvious. According to feline-specific antemortem diagnostic criteria proposed by Palerme et al. we found 1 major (positive echocardiographic findings) and 2 minor criteria (fever and microbiological evidence). Echocardiographic evaluation remains the reliable way to identify suspicious lesions and determine if further diagnostics should be performed [3]. The echocardiogram showed severe and widespread thickening of the aortic valve cusps and the aortic root (Figure 1); it was extremely indicative of an infective origin of the disease [2]. Advanced imaging and blood cultures are often required to confirm the antemortem diagnosis, but in our patient a singular urine culture allowed to isolate the causative agent. Positive urine culture is not a frequent finding in cats with infectious endocarditis [3, 16]. Conventional blood culture is not always useful and may also be difficult to obtain in feline patients because of the significant amount of blood needed to perform the test [3]. Conversely urine cultures of cystocentesis samples are easy to do and can be useful in cases of bacteremia and sepsis [17]. Unfortunately, we did not receive consent for blood culture, serology for FIV/FeLV and histological examination of the kidneys, therefore we were not allowed to evaluate if they were colonized, so it was not known if bacteriuria was associated with concurrent renal tissue infection. No microbiological examination of other organs (bowel, liver, and spleen) were carried out due to the owner's economic restriction. Significant bowel positivity would have clarified the pathogenesis of endocarditis. We assume, given the initial gastroenteric clinical signs, the intestine was the first organ involved in the infection and, after subsequent bacteremia, there was aortic valve and presumed kidney involvement. In a retrospective study of 29 cats with sepsis, myocardial necrosis and inflammation were found in nine cats, and bacteremia secondary to gastrointestinal tract disease were found in five of them [17]. For post-mortem diagnosis, according to the proposed modifications to the Duke criteria [18], we were able to confirm the suspected diagnosis of endocarditis and the pathological agent involved through histological examination [18, 17]. Bacteria are not always in valvular lesions, especially following antimicrobial therapy [2]. In our patient, despite the use of antibiotics, histological examination revealed suppurative endocarditis and myocarditis, highlighting the presence of bacterial aggregates (Figure 3). We found positivity and correspondence with what was detected in the myocardium, endocardium and a singular urine culture. Transient or persistent bacteremia is an absolute requirement for the establishment of a cardiac infection, but the onset episode of bacteremia is not always identified [17]. In acute infectious endocarditis, sepsis may cause focal or diffuse myocarditis and myonecrosis, colonies of bacteria can be detected in the myocardium in fulminant cases [2]. Salmonellosis is a disease of major zoonotic importance. The prevalence of Salmonellosis is difficult to establish, since the severity of clinical signs varies individually [19]. It is reasonable to wonder why *Salmonella *spp. colonized the endocardium of our patient. We know that Salmonella virulence involves multiple factors related to both the organism and the host [19]. Many pathogenic bacteria associated with infectious endocarditis have surface receptors for fibronectin [1, 2, 17]. Some *Salmonella typhimurium *strains showed the expression of SDHA-protein having fibronectin-binding activity [20]. We did not verify whether the strain of isolated *Salmonella typhimurium* expressed SDHA. Platelet-fibrin deposition on heart valves is hypothesized to be the scaffolding on which microorganisms can adhere and infiltrate; endothelial injury and hypercoagulable states could be the trigger mechanisms but the pathogenesis of infectious endocarditis is most likely multifactorial and not completely known [11, 21]. Key interactions between the infecting organism and the patient include the host's immune system response and the virulence of the same organism. Reduced or ineffective immune responses due to infection with feline leukemia virus, feline immunodeficiency virus, or prolonged steroid administration, have been postulated to play a part in bacterial adherence to the valvular structures in the heart [1]. Other risk factors that raise the clinical suspicion of endocarditis include infection of the gastrointestinal tract and urinary system [1]. We excluded prolonged administration of corticosteroids in our patient, but its immune status was unknown as the owner refused further investigations.

Considering outdoor activity, the owner was not sure that his cat had not fed on prey, food waste, and carrion, we supposed that the cat had eaten contaminated food. Given the patient's history, it is reasonable to assume that the origin of the infection was the gastrointestinal tract and that, after bacteremia, the aortic valve leaflet and myocardium were colonized. We confirmed the endocardial and myocardial localization of *Salmonella typhimurium *by histology, urine culture (*in vivo*), and aortic valve leaflet culture (*ex vivo*). Unfortunately we could not get further confirmation by polymerase chain reaction. Infectious endocarditis commonly carries a grave prognosis [3]. To the best of the authors' knowledge, this is the first report of *Salmonella typhymurium* endocarditis and myocarditis in a cat.
